# Supplementary material for: Frailty Assessment for Risk prediction in Gynecologic Oncology patients undergoing surgery and chemotherapy (FARGO) study protocol: Rationale and design of a multi-centre prospective cohort study
Source: PLoS One. 2025 Jul 28;20(7):e0325651. doi: 10.1371/journal.pone.0325651 (PMC12303337; doi:10.1371/journal.pone.0325651)
Supplement: S2 File — (PDF) [file pone.0325651.s002.pdf]

FARGO

FRAILTY ASSESSMENT FOR RISK PREDICTION IN GYNECOLOGIC ONCOLOGY PATIENTS

Frailty Assessment for Risk prediction in Gynecologic Oncology patients undergoing surgery and chemotherapy (FARGO study)

Protocol Final v5.0
Dated April 25, 2025

Sponsor and Study Coordinating Group:
FARGO Project Office
Population Health Research Institute
Hamilton General Hospital Campus, DBCVSRI
237 Barton Street East
Hamilton, Ontario, Canada L8L 2X2

Principal Investigators:
Drs. Maura Marcucci & Julie My Van Nguyen
Population Health Research Institute
DBCVSRI, 237 Barton Street East
Hamilton, Ontario, Canada L8L 2X2

Protocol Number: 2021.09.08
Clinicaltrials.gov Identifier: NCT05738252

This protocol is the confidential intellectual property of the FARGO Study Operations Committee, Population Health Research Institute. The use of any unpublished material presented in this document is restricted to the recipient for the agreed purpose and must not be disclosed to unauthorized persons without the written consent of the FARGO Study Operations Committee

STUDY SUMMARY

**Title**	**The FARGO Study**<br><br>Frailty Assessment for Risk prediction in Gynecologic Oncology patients undergoing surgery and chemotherapy
**Project Office**	Population Health Research Institute (PHRI)
**Study Size**	280 patients
**Study Design**	Multi-centre, prospective and longitudinal, cohort study
**Primary Objectives**	In patients aged 55 or older, with confirmed or suspected gynecologic cancer, undergoing cytoreductive or high-risk surgery with or without chemotherapy, to evaluate the performance of preoperative frailty assessment based on the Frailty Phenotype (FP), compared to a perioperative cardiovascular risk assessment based on the combination of preoperative Revised Cardiac Risk Index (RCRI), age and occurrence of myocardial injury after noncardiac surgery (MINS), in predicting the composite of all-cause death or new disability at 6 months after surgery.
**Secondary Objectives**	In patients aged 55 or older, with confirmed or suspected gynecologic cancer, undergoing cytoreductive or high-risk surgery with or without chemotherapy:

	1. to compare the predictive performance of *different preoperative/perioperative frailty assessments* for all-cause death or new disability at 6 months after surgery; the assessments will include:<br>a. different frailty tools, i.e., the whole FP, the single components of the FP, and the Clinical Frailty Scale (CFS); and<br>b. a *dynamic* perioperative frailty assessment (i.e., based on a frailty assessment repeated 28 days after surgery, in addition to a preoperative frailty assessment)<br>2. to explore the predictive performance of frailty assessments upon *chemotherapy-related outcomes*, including completion, total dose received, decisional regret, and impact on function;<br>3. to explore the value of frailty assessment *when added* to a perioperative cardiovascular risk assessment+age, with or without other clinical predictors, in predicting all-cause death or new disability at 6 months after surgery;<br>4. to explore the predictive performance of a preoperative frailty assessment and of a preoperative cardiovascular risk assessment based on RCRI+age upon *postoperative outcomes at 28 days after surgery*, including all-cause death or new disability; major vascular events; infection and sepsis; bleeding; new clinically relevant atrial fibrillation/flutter; acute congestive heart failure; length of stay; unplanned admission to intensive care unit; and delirium.<br>5. to explore the predictive performance of preoperative frailty versus a perioperative cardiovascular risk assessment based on RCRI +age upon all-cause death or new disability at 1 year after surgery;<br>6. to explore the predictive performance of a preoperative frailty assessment and of a perioperative cardiovascular risk assessment based on RCRI+age combined with MINS, upon other *long-term postoperative outcomes*, including major vascular events at 6 months and 1 year after surgery; infection and sepsis at 6 months and 1 year after surgery; and all-cause death at 6 months and 1 year after surgery.<br>7. to explore the predictive performance of a preoperative frailty assessment and of a perioperative cardiovascular risk assessment based on RCRI+age combined with MINS, upon *oncologic outcomes* of progression-free survival (PFS) and overall cancer-specific survival up to 1 year after surgery;<br>8. to measure *feasibility* and *acceptance* of frailty and disability assessments to patients and healthcare providers<br>9. to create a biobank to explore possible *biomarkers* of perioperative frailty
**Eligibility Criteria**	***Inclusion criteria***<br>1. Age must be 55 years or older at registration<br>2. Must meet any <u>one</u> of the following criteria:<br>a. Have stage II-IV ovarian or endometrial/uterine cancer, undergoing cytoreductive surgery via laparotomy, with or without neoadjuvant chemotherapy (NACT)<br>b. Have any stage endometrial, uterine or cervical cancer planned for laparotomy where laparoscopy is deemed unfeasible/high-risk due to comorbidities<br>c. Are undergoing laparotomy for pelvic mass, highly suspicious for malignancy; or<br>d. Are undergoing laparotomy for gynecologic malignancy recurrence.<br>***Exclusion criteria***<br>1. Unable to provide informed consent<br>2. Require urgent surgery within 24 hours of first consultation to the Gynecological Oncology team<br>3. Are undergoing neoadjuvant radiation therapy<br>4. Have a previously documented history of dementia<br>5. Have cognitive, language, vision, or hearing impairment that impacts ability to understand the directions for the completion of the study instruments<br>6. Are participating in a clinical trial investigating a new neoadjuvant systemic therapy
**Measurements**	Preoperatively, patients will have a frailty assessment (FP and CFS), Functional Assessment of Cancer Therapy – General – 7 Item Version (FACT-G7), and World

	Health Organization Disability Assessment Schedule 2.0 (WHODAS 2.0). For those patients undergoing NACT prior to surgery, they will have these assessments twice: once before and then again after their NACT (i.e., before surgery). All patients will have troponin measured on postoperative days 1, 2 and 3. In hospital, study personnel will administer the 3-minute diagnostic interview for the Confusion Assessment Method (3D-CAM) twice a day, during the first 3 days after surgery. Acceptance of frailty assessment according to physicians will be measured using the Ottawa Acceptability of Decision Rules Instrument (OADRI). Patient's acceptance of frailty and disability assessments will be evaluated in a sub-set of patients by qualitatively administering an exit interview. Blood collection for the optional biobank component will be completed before surgery, before and after NACT, at the 28-day post-surgery visit, 6-month post-surgery visit, and 1-year post-surgery visit.
**Follow-up**	Follow-up visits will be done by study personnel (either by phone, tele- or video-conference or in person) at 28 days, 6 months, and 1-year post-surgery. Efforts will be made to perform these visits in concomitance with clinical follow-ups. Frailty assessments will be repeated at the 28-day post-surgery visit. WHODAS 2.0 will be administered at the 28-day, 6-month and 1-year visits. FACT-G7 will be administered at the 28-day, 6-month and 1-year visits.

FARGO Protocol v5.0 Approval:

By signing the below, I designate my approval of the above-named version of the FARGO protocol.

Dr. M. Marcucci<br>Principal Investigator<br>Population Health Research Institute	Signed by:<br>Maura Marcucci<br>Signer Name: Maura Marcucci<br>Signing Reason: I approve this document<br>Signing Time: 2025-04-28 | 8:56:29 AM EDT<br>9C4C59821B5A447C93EA15FDD7AA179E<br>Signature	2025-04-28 | 8:56:46 AM EDT<br>Date (yyyy-mm-dd)

Dr. J.M.V. Nguyen<br>Principal Investigator<br>Population Health Research Institute	Signed by:<br>Julie My Van Nguyen<br>Signer Name: Julie My Van Nguyen<br>Signing Reason: I approve this document<br>Signing Time: 2025-04-25 | 7:51:01 AM PDT<br>CE5A0926C66A44F6905794F985A77FC4<br>Signature	2025-04-25 | 7:51:21 AM PDT<br>Date (yyyy-mm-dd)

LIST OF ABBREVIATIONS

3D-CAM	3-Minute Diagnostic Confusion Assessment Method
AUC	Area Under the Curve
CABG	Coronary Artery Bypass Graft
CAM	Confusion Assessment Method
CAM-ICU	Confusion Assessment Method for ICU Patients
CFS	Clinical Frailty Scale
CT Scan	Computed Tomography Scan
DVT	Deep Venous Thrombosis
ECG	Electrocardiogram
FACT-G7	Functional Assessment of Cancer Therapy – General – 7 Item Version
FARGO	Frailty Assessment for Risk prediction in Gynecologic Oncology patients undergoing surgery and chemotherapy Study
FP	Frailty Phenotype
GCP	Good Clinical Practice
GCS	Glasgow Coma Scale
GO	Gynecologic Oncology
hsTnT	high-sensitivity Troponin T
ICH	International Conference on Harmonisation
IEC	Independent Ethics Committee
IRB	Institutional Review Board
IWRS	Interactive Web Registration System
JHCC	Juravinski Hospital and Cancer Centre
LBBB	Left Bundle Branch Block
MINS	Myocardial Injury after Noncardiac Surgery
MRI	Magnetic Resonance Imaging
NACT	Neoadjuvant Chemotherapy
OADRI	Ottawa Acceptability of Decision Rules Instrument
OC	Operations Committee
OS	Overall Survival
PACU	Post-Anesthesia Care Unit
PCI	Percutaneous Coronary Intervention
PE	Pulmonary Embolism
PFS	Progression-Free Survival
PHRI	Population Health Research Institute
PI	Principal Investigator
PROs	Patient Reported Outcomes
QoL	Quality of Life
qSOFA	quick Sequential Organ Failure Assessment
RCRI	Revised Cardiac Risk Index
RDI	Relative Dose Intensity
ROC	Receiver Operator Characteristic
SC	Steering Committee
TnT	Troponin T
URL	Upper Reference Limit
VISION	Vascular events in Noncardiac Surgery Patients Cohort Evaluation
WHODAS 2.0	World Health Organization Disability Assessment Schedule 2.0

Contents

1 INTRODUCTION AND RATIONALE 7
1.1 Primary Research Question 7
1.2 Need for the FARGO Study 7
1.2.1 Current treatment pathways of patients with advanced or high-risk gynecologic cancer . 7
1.2.2 Current perioperative risk assessment in gynecologic oncologic patients and its limitations 8
1.2.3 Age-related frailty 8
1.2.4 Frailty in gynecologic oncologic patients undergoing surgery and systemic treatment: evidence and gaps 9
1.2.5 Feasibility and acceptability of frailty assessment and role of biomarkers 9
1.2.6 Summary of background and rationale 9
2 PLAN OF INVESTIGATION 10
2.1 Study Objectives 10
2.1.1 Primary objectives 10
2.1.2 Secondary objectives 10
2.2 Study Design 11
2.3 Sample Size 11
2.4 Eligibility Criteria 11
2.4.1 Inclusion criteria 11
2.4.2 Exclusion criteria 11
2.5 Patient Recruitment Plan and Informed Consent 12
2.6 Registration 12
2.7 Study flow 12
3. MAIN STUDY PREDICTORS (EXPOSURES) 12
3.1 Assessments 12
3.1.1 Frailty Phenotype 12
3.1.2 Clinical Frailty Scale 13
3.1.3 Revised Cardiac Risk Index (RCRI) and age 13
3.1.4 Preoperative RCRI+age, combined with MINS 13
3.2 Risk to the Safety of Patients 13
4. STUDY OUTCOMES 13
4.1 Primary clinical outcomes 14
4.2 Secondary clinical outcomes 14
4.3 Feasibility and acceptability outcomes 14
4.4 Adjudication of study outcomes 14
4.5 Minimizing bias 14
5. FOLLOW UP 15
5.1 Overall study 15
5.2 Biobank study 15
6. STATISTICAL ANALYSES 15
6.1 Primary Analysis 15
6.2 Secondary Analyses 16
7.0 SUB STUDIES 16
8.0 STUDY MANAGEMENT 16

8.1 Arrangements for the day to day management of the study 16
8.2 Steering and Publication Committees 16
8.3 Site Principal Investigators (PI) 17
9. ENSURING DATA QUALITY 17
9.1 Data Management 17
9.2 Confidentiality 17
10.0 ETHICAL CONSIDERATIONS 17
11. IMPORTANCE OF THE STUDY 18
Figures 18
Figure 1: Study Flow Diagram 18
Figure 2 - Study timeline, measurements and data collection 19
Appendices 20
Appendix I. Sample size calculations 20
Appendix II. Clinical outcomes definitions 20
Appendix III – Measuring acceptability of frailty assessment 25
References 27

1 INTRODUCTION AND RATIONALE

The increased age and comorbidities of our surgical population has been posing new challenges to the growing field of perioperative medicine. When older patients require surgery as a treatment for cancer, the complexity even increases. By 2030, 70% of all cancers will be diagnosed in adults aged 65 years and older [1]. This trend is also involving the Gynecologic Oncology (GO) population, with many GO malignancies diagnosed at an older age being at an advanced stage and/or requiring aggressive treatments. Even after adjusting for cancer stage, older age is associated with worse postoperative outcomes and disparities in treatment, including lower rates of initiation and completion of chemotherapy [2-4]. However, older GO patients can still show a good response to cytoreductive surgery. Age, comorbidities, and cancer stage alone seem insufficient to explain the complex prognosis of the GO population.

The concept of frailty has been used to define an age-related and disease-related state of vulnerability due to reduced capacity of different physiological systems. Frailty can be measured with questionnaires, physical tests, comprehensive clinical evaluations, or, potentially, through blood biomarkers. Measuring frailty might better capture the potential interaction between age, cancer, comorbidities, and treatment in GO patients in whom surgery and systemic treatment are considered.

1.1 Primary Research Question

How does preoperative frailty assessment based on the FP perform, compared to a perioperative cardiovascular risk assessment based on the combination of preoperative RCRI, age and occurrence of MINS, in predicting the composite of all-cause death or new disability at 6 months after surgery, in patients aged 55 or older, with confirmed or suspected gynecologic cancer, undergoing cytoreductive or high-risk surgery with or without chemotherapy?

1.2 Need for the FARGO Study

1.2.1 Current treatment pathways of patients with advanced or high-risk gynecologic cancer

Gynecologic malignancies include ovarian, endometrial/uterine, cervical, and vulvar cancers. Patients affected by these types of cancer may present at different stages of their disease and undergo different treatment pathways. When surgery is indicated, less invasive surgery procedures or techniques (e.g., laparoscopy) are preferred whenever possible, but they are not always feasible or appropriate. Neoadjuvant or adjuvant chemotherapy, and/or radiotherapy, could also be indicated based on stage.

Patients with ovarian cancer most often present at an advanced stage, requiring a combination of cytoreductive surgery via laparotomy and chemotherapy as standard treatment. Patients most often receive 6 cycles of chemotherapy. There are 2 possible sequences to treatment: a) Primary surgery protocol, which consists of primary cytoreductive surgery, followed by 6 cycles of adjuvant chemotherapy, or b) Neoadjuvant chemotherapy protocol, which consist of 3 to 4 cycles of chemotherapy, followed by interval cytoreductive surgery, followed by another 3 cycles of chemotherapy. The decision regarding the sequence of treatment is made by the treating gynecologic oncologist, and largely depends on tumor distribution and patient performance status. There is evidence that primary cytoreductive surgery has improved oncologic outcomes, in the selected patient population: patients undergoing primary surgery have a survival benefit of years compared to those undergoing interval surgery.[5,6] Patients who have poor performance status and several comorbidities are typically assigned to the neoadjuvant protocol.

Patients with endometrial/uterine cancer or cervical cancer are often diagnosed by their general gynecologist or family physician, then referred to a Gynecologic Oncologist. At the initial consultation, the Gynecologic Oncologist will order staging investigations (Computed Tomography Scan [CT Scan] and/or Magnetic resonance imaging [MRI]) and decide based on the patient's general performance status whether they are a candidate for surgery. Surgery is typically performed within 4 to 6 weeks of the initial consultation. Oncologic reasons and presence of comorbidities precluding laparoscopy drive the decision between laparotomy and laparoscopy. Based on the final pathology from surgery, adjuvant chemotherapy and/or radiation therapy may be recommended.

Overall, treatment pathways for advanced or high-risk gynecologic cancer can include aggressive therapies associated with possible complications and side effects. These treatments have proved effective in improving patient outcomes. Decision regarding surgery and chemotherapy

candidacy, sequence, and regimens, can have a significant impact on compliance to treatment, short-term morbidity, and long-term survival.[7,8] Treatment delays, for example in initiation of chemotherapy, can be associated to decreased survival.[8] Hence, at several time points through a patient's GO journey, an individualized assessment of treatment-associated risks becomes cornerstone in guiding and tailoring treatment, in order to minimize complications, and optimize oncologic outcomes and QoL.

1.2.2 Current perioperative risk assessment in gynecologic oncologic patients and its limitations

In current practice, decisions regarding surgery and chemotherapy candidacy are initially made by the treating gynecologic oncologist based on a subjective judgement of the expected treatment tolerance, based on a non-systematic assessment of patient performance status, and most often influenced by the patient chronologic age.[9] When a patient is deemed to be a probable surgical candidate, anesthesia, internal medicine, cardiology, and/or perioperative medicine consultants are often involved, based on patient age and/or presence of comorbidities, to finalize the decision about surgery and optimize the perioperative risk management. Cardiovascular risk assessment often plays a major role in this perioperative risk evaluation.

The RCRI is currently the most validated index among the clinical risk indexes for preoperative cardiac risk assessment. The RCRI includes 6 factors, each worth 1 point (i.e., history of ischemic heart disease, cerebrovascular disease, congestive heart failure, preoperative insulin use, preoperative creatinine > 177 mmol/L, and high-risk surgery).[10] In a systematic review that included 792,740 patients from 24 studies, the RCRI showed moderate discrimination to predict major perioperative cardiac complications.[11] The Canadian Cardiovascular Society Guidelines on Perioperative Cardiac Risk Assessment and Management for Patients Who Undergo Noncardiac Surgery suggest clinicians use the RCRI over the other available clinical risk prediction scores (Conditional Recommendation; Low-Quality Evidence).[12] These guidelines also provide updated pooled risk estimates obtained from external validation studies of the RCRI that were published in the past 15 years, in which perioperative troponin was systematically monitored.[12]

The VISION study, a prospective cohort study of 40,000 patients aged ≥45 years-old undergoing noncardiac surgery worldwide, has showed that MINS, defined as a postoperative troponin elevation judged to be due to myocardial ischemia, with or without symptoms or electrocardiogram (ECG) changes, is associated with a >3 fold increase in any-cause mortality within 30 day from surgery, with the highest attributable risk for death (34%) among all major postoperative complications.[13-15] MINS is also strongly associated with mortality at 1 year.[16] Embedding this evidence, the Canadian guidelines recommend a preoperative cardiovascular risk stratification that starts with calculating the RCRI; in those with a RCRI ≥1, or with a RCRI=0 but 65 years old or older, troponin monitoring for the first 3 days after surgery is recommended.[12]

These perioperative risk guidelines are followed in many centres in Canada and worldwide. However, this approach might have limitations in the GO population. Unpublished confidential data from VISION show that, among 271 women aged 55 or older undergoing major surgery for urologic or gynecologic cancers, MINS was associated with a 2-to-3-fold increase in mortality at 1 year; however, those with no MINS (and not meeting the RCRI+age criteria for high risk) still had a 1-year mortality of 14% (compared to 3% in same age women in the overall VISION cohort without MINS). Limitations of the VISION study in order to inform our current practice with GO patients include: poor representation of this population; no collection of data on cancer-specific risk factors, and on preoperative and postoperative chemotherapy; no predictive ability for non-cardiac postoperative morbidity, and no intermediate time points (i.e., >30 days and <1 year). In addition, VISION did not include the assessment of patient reported outcomes (PROs), like QoL, functional recovery, or disability, which are poorly studied in the GO literature, but might be the most relevant to patients.

At no time does the current perioperative assessment of GO patients include frailty.

1.2.3 Age-related frailty

Frailty is being increasingly studied as predictor of adverse outcomes. Frailty is a multidimensional geriatric syndrome characterized by increased vulnerability to stressors as a result of reduced capacity of different physiological systems.[17,18] Cumulative evidence has associated frailty with an increased risk of adverse health-related outcomes including falls, disability, hospitalizations and mortality.[19,20] In patients who undergo surgery, frailty is associated with increased short-term morbidity, including falls, disability, increased resource utilization, and 1-year mortality [21-25] after

surgery. In patients living with cancer, frailty may also independently predict oncologic outcomes [26,27] and treatment tolerance[28]. Several ways of operationalizing this concept have been proposed, without reaching a formal consensus.[20,29-32] Two aspects, however, are common across different frailty definitions. First, they have been consistently associated with adverse outcomes. Second, although frailty is often described as being present or absent, frailty indexes or scales can be used to grade a condition with higher levels of frailty being associated with greater vulnerability and risk of adverse outcomes.

The most commonly used definition of frailty was developed by Fried et al. as part of their work in the Cardiovascular health study and the Women's Health and Aging Studies.[29] It has a focus on the physical domain and it is known as Physical Frailty or FP. According to this definition, frailty corresponds to a condition meeting at least 3 out of 5 criteria, including weak muscle strength, slow gait speed, unintentional weight loss, exhaustion, and low physical activity.

Many investigators have suggested a broader multidomain framework for frailty. According to the accumulating deficit model, for example, frailty is measured as an index obtained by counting the number of deficits present across multiple domains (≥30 deficits must be assessed).[20,30]

1.2.4 Frailty in gynecologic oncologic patients undergoing surgery and systemic treatment: evidence and gaps

Frailty is being increasingly studied as a predictor of adverse outcomes in older adults after surgery. Large patient cohort and population-based studies in noncardiac surgeries have showed the association of frailty, measured by questionnaires, physical performance tests, clinician judgment, or administrative data, with postoperative mortality, disability, institutionalization, and resources utilization. However so far these studies have not included any or only selected (i.e. hysterectomies) surgical GO patients, and no comparison with other types of perioperative risk assessments has been done.

There are very few studies on frailty in the GO literature. Overall, they suggest that frailty can affect cancer-specific outcomes and treatments (e.g. tolerance to chemotherapy[3]), and overall prognosis[39-43]. However, these studies are small and/or in specific cancer groups, and with methodological limitations. Also, none of the existing studies has considered the complex and dynamic treatment trajectories of these patients, including surgery but often also neoadjuvant and adjuvant chemotherapy, which points at the possibly greater utility of a dynamic versus static (i.e. one-time) frailty assessment. Using the JHCC National Surgical Quality Improvement Program database, our team identified that in a population of over 250 GO patients undergoing a laparotomy, frailty was significantly associated with 30-day severe postoperative morbidity, increased length of stay, non-home discharge, and inability to complete adjuvant chemotherapy.[44]

While there is mounting evidence in other surgical fields that frailty impacts short and long-term surgical and oncologic outcomes, the decisions regarding candidacy for surgery and/or chemotherapy in GO do not currently employ standard frailty assessments despite the increasingly complex and aging patient population.

1.2.5 Feasibility and acceptability of frailty assessment and role of biomarkers

The feasibility of measuring frailty (e.g. time and resource required) is a known barrier to the adoption of frailty assessment in clinical practice, especially in busy clinical settings. In particular, GO patients, especially if old and ill, might not perceive the benefits of an additional assessment that requires time and/or the performance of physical tests and/or questions that they might find difficult to answer. This highlights the clinical relevance of studying biomarkers for perioperative frailty, i.e., biomarkers whose levels of expression specifically (dynamically) change with frailty as clinically-assessed, and which could potentially replace clinical assessments. With the advance of point of care devices that can also measure gene and gene expression, different types of biomarkers could be available in real time for decision-making. For example, mitochondrial DNA copy number (mtDNA-CN), a measure of mtDNA levels per cell, declines with age, and can be measured in peripheral blood leukocytes with a low-cost scalable assay. Low mtDNA-CN is a marker of mitochondrial dysfunction, energy reserves, oxidative stress, and systemic inflammation[45]. mtDNA is a promising biomarker that has been correlated with frailty and adverse outcomes[45-48].

1.2.6 Summary of background and rationale

Little is known about how to best predict postoperative outcomes, recovery from complications, and chemotherapy tolerance in an increasingly older and medically complex GO population. Measuring frailty may represent a comprehensive tool for risk prediction. Our study 1) will help fill the current gap in knowledge; 2) will translate into clinical practice changes both locally, and when replicated in a larger multi-centre study, elsewhere in Canada and worldwide; and 3) will inform future studies on shared decision-making strategies and interventions.

By evaluating the role of frailty as a static or dynamic predictor of patient important outcomes, and by considering the complexity of these patients and also of their treatment trajectories, our study has the potential to fill those gaps and influence how care is delivered. By involving stakeholders in the evaluation of feasibility and acceptability of frailty assessment, this will inform a change in care that is sustainable, innovative, and patient-centred.

There have been substantial knowledge advancements about perioperative risk factors and the long-term impact of postoperative complications; however, oncology patients and patient-reported outcomes have been insufficiently studied. There is increased literature on frailty assessment in noncardiac surgery; however, studies that included GO patients are few and of low-quality. Our study will overcome the limitations of the current knowledge and practice, and will potentially change healthcare delivery.

2 PLAN OF INVESTIGATION

2.1 Study Objectives

2.1.1 Primary objectives

To evaluate the performance of preoperative frailty assessment based on the FP, compared to a perioperative cardiovascular risk assessment based on the combination of preoperative RCRI, age and occurrence of MINS, in predicting the composite of all-cause death or new disability at 6 months after surgery, in patients aged 55 or older, with confirmed or suspected gynecologic cancer, undergoing cytoreductive or high-risk surgery with or without chemotherapy.

2.1.2 Secondary objectives

In patients aged 55 or older, with confirmed or suspected gynecologic cancer, undergoing cytoreductive or high-risk surgery with or without chemotherapy:

1. to compare the predictive performance of *different preoperative/perioperative frailty assessments* for all-cause death or new disability at 6 months after surgery; the assessments will include:
    a. different frailty tools, i.e., the whole FP, the single components of the FP, and the CFS; and
    b. a *dynamic* perioperative frailty assessment (i.e., based on a frailty assessment repeated 28 days after surgery, in addition to a preoperative frailty assessment)
2. to explore the predictive performance of frailty assessments upon *chemotherapy-related outcomes*, including completion, total dose received, decisional regret, and impact on function;
3. to explore the value of preoperative frailty assessment *when added* to a perioperative cardiovascular risk assessment+age, with or without other clinical predictors, in predicting all-cause death or new disability at 6 months after surgery;
4. to explore the predictive performance of a preoperative frailty assessment and of a preoperative cardiovascular risk assessment based on RCRI+age upon *postoperative outcomes at 28 days after surgery*, including all-cause death or new disability; major vascular events (as composite of vascular death, and nonfatal myocardial injury, stroke, symptomatic proximal venous thromboembolism, and cardiac arrest); infection and sepsis; Bleeding Independently Associated with Mortality after noncardiac Surgery (BIMS), acute congestive heart failure, new clinically significant atrial fibrillation/flutter; length of stay; unplanned admission to intensive care unit; and delirium;
5. to explore the predictive performance of preoperative frailty versus a perioperative cardiovascular risk assessment based on RCRI +age upon all-cause death or new disability at 1 year after surgery;
6. to explore the predictive performance of a preoperative frailty assessment and of a perioperative cardiovascular risk assessment based on RCRI+age combined with MINS, upon other *long-term postoperative outcomes*, including major vascular events at 6 months and 1 year after surgery; infection and sepsis at 6 months and 1 year after surgery; and all-cause death at 6 months and 1 year after surgery.

7. to explore the predictive performance of a preoperative frailty assessment and of a perioperative cardiovascular risk assessment based on RCRI+age combined with MINS, upon *oncologic outcomes* of PFS and overall cancer-specific survival (after surgery) up to 1 year after surgery;
8. to measure *feasibility* and *acceptance* of frailty and disability assessments to patients and healthcare providers;
9. to create a biobank to explore possible *biomarkers* of perioperative frailty.

2.2 Study Design

This is a multicentre prospective cohort study of patients 55 years old or older with confirmed or suspected gynecologic cancer who are candidates to cytoreductive or high-risk surgery (laparotomy) with or without chemotherapy at centres.

2.3 Sample Size

The FARGO study is sized to respond to the primary objective. We expect a 20% rate of death or new disability at 6 months after surgery (primary outcome). We performed preliminary analyses of the VISION cohort, where data for women undergoing surgery for gynecologic cancer could not be distinguished from women undergoing surgery for urological cancer. Based on these analyses, we expect that, in our study population, the ability of the baseline RCRI+age combined with the occurrence of MINS to predict 6-month death or new disability, measured as Area Under the receiver operating characteristic Curve (AUC), will not be greater than 0.600 (range 0.550-0.600, Appendix I). A sample size of 280 patients will give us at least 80% of power to detect a difference of at least 0.130 in the AUC for a model including only the FP-frailty assessment, compared with the AUC for the model including preoperative RCRI+age combined with MINS (i.e., AUC for FP-frailty model of at least 0.680[21]). The table in the Appendix I shows the sample size calculation based on these assumptions for a range of scenarios, based on the comparison of 2 Receiving Operating Characteristic (ROC) curves, 2-sided test, alpha=0.05, and assumed correlation between the 2 predictive models=0.5.

We will include 280 patients in order to conservatively account for ≤ 10% of participants who will be lost before or during surgery, and in whom troponin measurement to assess for MINS will not be possible (in fact, we expect that after recruitment much fewer patients will not undergo or survive surgery). Based on our eligibility criteria, we expect that at least 50% (i.e., 140) of our participants will undergo chemotherapy, before and or after surgery, and will be included in our exploratory secondary analyses on chemotherapy outcomes.

2.4 Eligibility Criteria

2.4.1 Inclusion criteria

Patients are eligible if they are 55 years or older at the time of registration AND meet any one of the following criteria:

1. have stage II-IV ovarian or endometrial/uterine cancer, undergoing cytoreductive surgery via laparotomy, with or without NACT
2. have any stage endometrial, uterine or cervical cancer planned for laparotomy where laparoscopy is deemed unfeasible/high-risk due to comorbidities
3. are undergoing laparotomy for pelvic mass, highly suspicious for malignancy; or
4. are undergoing laparotomy for gynecologic malignancy recurrence.

2.4.2 Exclusion criteria

Patients are ineligible if they meet any one of the following criteria:

1. are unable to provide informed consent
2. require urgent surgery within 24 hours of first consultation to the GO team
3. are undergoing neoadjuvant radiation therapy
4. have a previously documented history of dementia
5. have cognitive, language, vision, or hearing impairment that impacts ability to understand the directions for the completion of the study instruments
6. are participating in a clinical trial investigating a new neoadjuvant systemic therapy

2.5 Patient Recruitment Plan and Informed Consent

At least 3 centres will participate in the FARGO study. Research personnel will screen the list of patients aged 55 years or older, diagnosed with a suspected or confirmed gynecologic malignancy and requiring a) a surgical intervention performed by laparotomy or b) NACT, with plan for interval surgery via laparotomy, who are scheduled for any pre-operative or pre-chemotherapy visit at the participating centres. Clinics involved in the pre-operative or pre-chemotherapy assessment of the potential study population as per local practice (e.g., GO clinics, internal medicine clinics, anesthesiology clinics, etc.) will be engaged in the patient screening process. If a patient meets the criteria, study personnel will approach the patient to obtain their informed consent. Based upon the clinic schedule and access to the patients, a combination of remote and written consent may be used based upon the patient preferences. If the patient chooses remote consent, the signed ICF will be returned to the site prior to initiation of study procedures. These informed consent options may be provided to reduce burden on the patient population.

2.6 Registration

Registration will occur after a patient is deemed eligible. Informed consent is obtained. For patients who are not receiving chemotherapy before surgery, registration should occur within 45 days prior to their surgery. For patients who are receiving chemotherapy before surgery, registration should occur within 45 days prior to their first chemotherapy treatment. Research personnel will register patients via a password protected Interactive Web Registration System (IWRS). The IWRS is a 24-hour computerized registration internet system maintained by the coordinating centre at the PHRI, which is part of Hamilton Health Sciences and McMaster University in Hamilton, Ontario, Canada.

2.7 Study flow

Figure 1 shows the FARGO patient flow diagram. We expect that some of the eligible patients (group A) will undergo surgery without NACT. Group A will undergo only one preoperative study visit (baseline) within 45 days prior to their surgery. Patients undergoing NACT before their surgery (group B) will have a first baseline study assessment within 45 days prior to their first chemotherapy treatment; in this group, a second preoperative study visit will be repeated after chemotherapy is considered terminated at least 18 days post-chemotherapy cycle and within 45 days prior to surgery. Post-chemotherapy, a small proportion of patients will not be deemed eligible for surgery by the treating physician (group C). Group C will be included in the evaluation of chemotherapy-related outcomes and will also be asked to complete a follow-up visit 6 months from the date of their registration. We expect an even smaller proportion of patients to be initially deemed eligible, recruited, and who will complete the baseline assessment; however, they will eventually not undergo any surgery nor chemotherapy (Group D, not represented in Figure 1). For Group D, a follow-up visit at 6 months from the baseline visit will be completed. Group A and B will complete the study assessments and follow-ups. Some patients of group A and group B will undergo adjuvant chemotherapy after surgery; this will not affect their study timeline.

3. MAIN STUDY PREDICTORS (EXPOSURES)

3.1 Assessments

We will assess every patient enrolled upon the following predictors.

3.1.1 Frailty Phenotype

Frailty will be measured based on the FP evaluation.[29] The FP assessment is based on the following 5 criteria: weak muscle strength (decreased grip strength measured with a dynamometer), slow gait speed (measured on the 15-ft walking test), unintentional weight loss (> 10 lbs), exhaustion (self-reported), and low physical activity (low weekly energy expenditure). The patient receives 1 point for each criterion met, for a total score of: 0 to 1, not frail; 2 to 3, intermediate frail (pre-frail); 4 to 5, frail. The frailty status based on these categories will be evaluated as exposure for the primary research question. The actual FP score (i.e., from 0 to 5) and the score upon each of the components of the FP assessment will be secondarily evaluated as exposures/predictors. With an exploratory intent, at the first study FP assessment, the level of physical activity will be evaluated with regards to two different time point, i.e., the current level (which is what will enter the FP scoring for the purpose of the study

objectives), and the usual level (before the diagnosis of cancer was made, or before the cancer likely started affecting their performance and lifestyle).

3.1.2 Clinical Frailty Scale

Frailty will be also assessed using the Clinical Frailty Scale (CFS).[30] The CFS is a global frailty scale according to which the patient is assigned a category from 1 (“Very fit”) to 9 (“Terminally ill”), as a summative clinical judgment; frailty is present if a category >=4 is assigned. Depending on the study objective, the FP-based or CFS-based assessment will enter the predictive models either as preoperative baseline (static) variable only, or as a dynamic predictor obtained by a repeated postoperative assessment to reflect the impact of NACT, when applicable (i.e., change in frailty from before to after NACT but before surgery); and the impact of surgery (i.e., change in frailty from preoperative to 28 days after surgery).

3.1.3 Revised Cardiac Risk Index (RCRI) and age

Based on the collection of data on the past medical history at the time of undergoing surgery and on the type of surgery, each participant will have a preoperative RCRI calculated.[49] The RCRI includes 6 factors, each worth 1 point (i.e., history of ischemic heart disease, cerebrovascular disease, congestive heart failure, preoperative insulin use, preoperative creatinine > 177 mmol/L, and high-risk surgery). For the purpose of the primary research question, each participant will be classified as being at high risk if their RCRI ≥1, or if the RCRI is 0 but they are 65 years old or older. Secondarily, the predictive performance of the RCRI as a 0-to-6 score (combined or not with age) will be evaluated.

3.1.4 Preoperative RCRI+age, combined with MINS

For the purpose of the study, and to reflect the current practice, MINS will be defined as any elevated troponin (higher than the local lab threshold) judged to be due to myocardial ischemia (i.e., without evidence of a non-ischemic etiology [e.g. chronic elevation like in chronic heart failure, pulmonary embolism {PE}, sepsis, cardioversion]) that occurred within the first 28 days after the initiation of surgery. *The only exceptions to the definition of an elevated troponin will be to use a higher threshold for troponin T (TnT) of ≥30 ng/L, and for high-sensitivity troponin T (hsTnT) of 20 to <65 ng/L with an absolute change of at least 5 ng/L or an hsTnT level ≥65 ng/L. These threshold for TnT and hsTnT are based upon data from a large international prospective perioperative cohort study that established troponin thresholds that were independently associated with 30-day mortality after noncardiac surgery.* The occurrence of MINS according to this definition, as a predictor/exposure, will be based on the in-hospital postoperative troponin measurement, and on any other troponin measurement occurring within the 28 days after surgery. For the primary research question, patients will be considered as meeting the MINS definition regardless of whether this meets the universal definition of myocardial infarction.

For the purpose of the primary research questions, study participants will be assigned to the high-risk category (i.e., ‘exposed’) when they are at high risk as per the preoperative RCRI+age assessment and they experience MINS; and to the low-risk category (i.e., not ‘exposed’), if they are at high risk as per the preoperative RCRI+age assessment but they do not experience MINS, or if they are at low risk as per the preoperative RCRI+age assessment.

3.2 Risk to the Safety of Patients

It is unlikely that there are risks associated with completing the frailty assessments via the FP and CFS tools. The risk of loss of confidentiality to study participants is minimal, as all the electronic data will be deidentified, and will be stored in password-protected files in password-protected computers. The study key will be locked in cabinet, in a locked office.

Potential benefits include early recognition of lower functional status and frailty, which may not otherwise have been detected through routine preoperative assessment. This could prompt early intervention (for example, a referral to a physiotherapist and/or dietician prior to surgery).

4. STUDY OUTCOMES

The predictive performance of perioperative frailty and cardiovascular risk assessments will be assessed upon the following clinical outcomes.

4.1 Primary clinical outcomes

The primary outcome is all-cause death or new disability at 6 months after surgery. We will measure disability in several domains (including cognition, mobility, self-care, participation) using the 12-item World Health Organization Disability Assessment Schedule (WHODAS 2.0), a patient-reported outcome tool widely validated in surgical older populations. New disability will be defined as a disability score >=25% at follow-up, or a score increase of >=8% for those already disabled at baseline.[50]

4.2 Secondary clinical outcomes

Secondary outcomes include:

- all cause death or new disability at 28 days and at 1 year after surgery;
- all cause death at 28 days, 6 months, and 1 year after surgery;
- oncologic outcomes, including progression-free survival (PFS) and cancer-specific death, up to 1 year after surgery;
- major vascular complications (at 28 days, 6 months, and 1 year after surgery) defined as a composite of vascular death, and non-fatal myocardial infarction (or myocardial injury for the 28-day time point), stroke, symptomatic proximal venous thromboembolism, and cardiac arrest;
- Bleeding Independently Associated with Mortality after noncardiac Surgery (BIMS) at 28 days after surgery;
- new clinically important atrial fibrillation at 28 days, 6 months, and 1 year after surgery;
- acute congestive heart failure at 28 days, 6 months, and 1 year after surgery;
- infection, and infection with sepsis (at 28 days, 6 months, and 1 year after surgery);
- unplanned admission to ICU (during the index hospital admission for surgery);
- length of stay (during the index hospital admission for surgery);
- in-hospital delirium (during the index hospital admission for surgery);
- chemotherapy-related outcomes, which include: tolerance as objectively defined by total dose received and time to completion; patient’s decisional regret (i.e., distress or remorse after a health care decision, i.e., to undergo chemotherapy); change in health-related function (WHODAS 2.0); and change in health-related quality of life (FACT-G7);.

Appendix II includes the study clinical outcome definitions.

4.3 Feasibility and acceptability outcomes

We will collect time and rate of completion of the FP and CFS (and sub-items of the FP). Acceptance of frailty assessment according to physicians will be defined based on ease of use and clinical relevance, according to relevant items of the OADRI.[51] Patient’s acceptability of frailty and disability assessments will be measured administering a brief exit questionnaire. Appendix III includes details on how acceptability of frailty instruments from physicians’ and patients’ perspective will be measured in the study.

4.4 Adjudication of study outcomes

The Event Adjudication Committee consists of clinicians with expertise in perioperative outcomes who are blinded to treatment allocation and who will oversee the adjudication of the following outcomes: MINS and MI. We will use the decisions of the adjudication process for all statistical analyses of these events. An event adjudication plan will be developed and govern all details, definitions, and processes of this committee.

4.5 Minimizing bias

In order to reduce the influence from the knowledge of the participant frailty status (predictor) on the evaluation of disability (outcome), whenever possible WHODAS 2.0 will be administered by different study personnel from those who administered the frailty assessments. In this way, they will be able to be blinded to the patient RCRI.

5. FOLLOW UP

5.1 Overall study

Figure 2 shows the study participant timeline.

At baseline (i.e., before surgery, or before NACT for some cases), trained research personnel will collect relevant data about participant demographics, clinical and social history; will administer the WHODAS 2.0 questionnaire, and FACT-G7; and will assess for frailty. A trained clinical research assistant will measure frailty, first based on the CFS, and then administering the FP tests/questions. In patients undergoing NACT, the study personnel will repeat the frailty and WHODAS 2.0 assessments before surgery, after the NACT is deemed to be terminated.

Study personnel will follow patients throughout their time in hospital for surgery, reviewing their medical records and recording any clinical outcome. In hospital, all patients will have troponin measured on the 1st, 2nd, and 3rd day after surgery or until discharge, whichever comes first. Data on ischemic symptoms/signs will be collected, and an ECG will be performed if an elevated troponin measurement is detected. Data on any other troponin measurement that will be undertaken in any study participant within 28 days from surgery will be also collected and will be assessed upon the study definition for MINS. In hospital, study personnel will administer the 3D-CAM twice a day, during the first 3 days after surgery or until discharge, whatever comes first. We will use the CAM-ICU while the patients are in the Post-Anesthesia Care Unit (PACU) or if sent to ICU.

A follow-up study visit will be done at 28 days, 6 months, and 12 months after surgery. Efforts will be made to perform such study visits in concomitance with clinical follow-ups. Frailty assessments will be repeated at 28 days. WHODAS 2.0 will be administered at 28 days, 6 months and 12 months, in-person or over the phone, by research personnel blinded from to the frailty assessments. FACT-G7 will be administered at 28 days and 6 and 12 months (in-person or through videoconference). Study personnel will also collect clinical outcome data through the study follow-ups.

We will also assess physicians' and patients' acceptability of frailty assessments (Appendix III).

A patient engagement component will also be included. Patient engagement will be refined throughout the study with input from the patient engagement members according to Patient Engagement Integrated Knowledge Translation Committee Charter. At least two community members will be invited to join to create a viable patient engagement and integrated knowledge translation plan that will meet instrumental and strategic targets as defined by the committee.

5.2 Biobank study

Only participants providing specific separate consent will be enrolled in the biobank study. We will collect blood samples from patients at baseline, i.e., before and after starting the NACT and/or before surgery, and then at 28 days after surgery, 6 months and 12 months. We will collect EDTA tubes to obtain both plasma and whole blood for DNA analysis. Samples will be stored at –160°C in liquid nitrogen vapour.

6. STATISTICAL ANALYSES

6.1 Primary Analysis

The primary analysis will compare the performance of preoperative frailty assessment based on the FP and perioperative cardiovascular risk assessment based on the combination of preoperative RCRI, age and occurrence of MINS, in predicting the composite of all-cause death or new disability at 6 months after surgery. We will assess the association of FP and of RCRI+age+MINS with the primary outcome using logistic regression models. We will perform one logistic regression model including frailty as predictor, and one logistic regression model including the baseline RCRI+age+MINS as predictors. For each model we will then calculate the c-statistics (corresponding to the AUROC) and compare them using a non-parametric approach.[52] We will include in the primary analysis only study participants who are alive at 28 days after surgery. We expect this will exclude from the primary analysis a very small proportion of patients initially enrolled in the study (i.e., ≤2%). We will initially exclude from the primary analysis those patients for whom postoperative troponin measurement was not performed (for reasons other than death); as a sensitivity analysis, these patients will be then

included and considered as not having MINS. For the primary analysis we will not include other covariates in the regression models. We will secondarily include the type of gynecological diagnosis/indication (based on our inclusion criteria) as covariate and as effect modifier.

As part of our secondary objectives, we will also evaluate the utility of frailty *when added* to the RCRI+age+MINS as predictors by calculating the net absolute reclassification improvement.[53]

6.2 Secondary Analyses

To compare the predictive performance of different frailty assessments, we will use a similar approach as for the primary outcome, i.e., we will compare the c-statistics of logistic regression models, each including one type of frailty assessment.

To examine the added value of a dynamic perioperative frailty assessment on the primary outcome, we will use a logistic regression model including as predictor with preoperative frailty and change in frailty at 28 days after surgery (either as absolute change and as percent change).

Logistic regression or time-to-event models will be performed for the association of frailty and perioperative cardiovascular risk assessment with other outcomes, including chemotherapy-related outcomes, as appropriate.

Effects sizes (odds ratios or hazard ratios) will be presented together with 95% confidence intervals. The threshold for statistical significance will be set as $p<0.05$.

The physicians' and patients' acceptability scores will be compared between frailty instruments using Wilcoxon signed rank tests. The mean and standard deviation (in seconds) required to complete each frailty instrument will compared using Student t tests; the numbers of missing values will be compared using 2x2 tests.

7.0 SUB STUDIES

Sub-studies may be added at a later date based on the recommendation of the Project Office Operations Committee. These sub-studies are not part of the main protocol and the analysis and reporting of these sub-studies will be separate from the main study.

8.0 STUDY MANAGEMENT

8.1 Arrangements for the day to day management of the study

The PHRI Project Office is the coordinating centre for this study and is responsible for the development of the protocol, development of the enrollment programming, study database, data consistency checks, data analyses, coordination of the study centres, and conducting the study. The Co-Principal Investigators (co-PIs), Project Officer, Program Manager, and Research Coordinator are responsible for the activities of the Project Office. No statistician with knowledge of the study's protocol will participate in the management or coordination of the FARGO study. Figure 1 describes the proposed phases and associated timelines of the FARGO study. The study will end when all the recruited patients are actively followed for 1-year.

8.2 Steering and Publication Committees

The Steering Committee (SC) will meet at least once if needed during the planning, recruitment, and close-out phases of the study, and at any other time point as deemed necessary by the Chair or study Co-PIs. The SC will provide oversight of all study-related activities and will provide input into protocol development and any revisions, and review all aspects related to study conduct including recruitment rates, adherence rates, completeness of follow-up, and data quality; and to provide recommendations to address concerns. The SC will appoint members to a Publication Committee. This committee will create guidelines for publications related to FARGO. Publications will be authored by specific individuals on behalf of the FARGO Investigators. Individuals selected to

lead the writing of these publications will depend on their role in and contribution to FARGO, scientific interest, and scientific expertise.

8.3 Site Principal Investigators (PI)

All participating centres will have a site PI, and this individual is responsible for ensuring compliance with respect to the intervention, visit schedule, and procedures required by the protocol. The site PI will ensure the provision of all information requested in the data collection forms in an accurate and timely manner according to instructions provided. The site PI will maintain patient confidentiality with respect to all information accumulated during the study, other than that information to be disclosed by law.

9. ENSURING DATA QUALITY

9.1 Data Management

Study personnel will complete data collection forms through a program called TrialMaster. Data will be stored on a secure server located at PHRI. Source documentation supporting the information reported on the data collection forms will be filed at the site and made available for monitoring, Institutional Review Board/Independent Ethics Committee (IRB/IEC) review, and regulatory inspections, when required. The Investigator must retain all study records/files in accordance with applicable regulatory requirements. The Data Management Plan will outline the procedures to ensure data quality and will include the following:

1. All research personnel will undergo a training session before study commencement to ensure consistency in study procedures including data collection and reporting
2. All centres will have a detailed study Manual of Operations that will outline each step of the protocol
3. The Project Office personnel will review detailed monthly reports on screening, enrollment, patient follow-up, data transmission, thoroughness, and completeness of data collection, and event rates, and they will rapidly address any identified issues
4. The programmer will create internal validity and range checks using Trial Master which will identify any errors or omissions and notify the sender and Project Office of any such issues
5. The Project Office will undertake data validation of the study data collection forms
6. The Project Office will send investigators regular quality control reports
7. The study statistician will undertake statistical monitoring to identify outliers

9.2 Confidentiality

All patient information will be stored on a secure server and kept strictly confidential. Patients will be assigned a unique study identification number to ensure anonymity. These ID numbers will correspond to allocation data in secure electronic files.

Centres will be instructed to store any patient information containing names or other personal identifiers separately and identified by their ID number. Centres will secure all local databases with password-protected access systems. Individual subject medical information obtained as a result of this study is considered confidential and disclosure to third parties is prohibited except for the following reason. Medical information may be given to the subject’s personal physician or to other appropriate medical personnel responsible for the subject’s welfare.

Data generated as a result of the study are to be available for inspection on request by the participating physicians, IRB/IEC, and regulatory authorities.

10.0 ETHICAL CONSIDERATIONS

This study will be conducted in compliance with the protocol, principles laid down in the Declaration of Helsinki, Good Clinical Practice (GCP), as defined by the International Conference on

Harmonisation (ICH), and all applicable laws and regulations. Before study initiation, the site PI must have written and dated approval/favorable opinion from the IRB/IEC for the protocol and consent form. Amendments to the protocol will require IRB/IEC approval.

11. IMPORTANCE OF THE STUDY

Little is known about how to best predict postoperative outcomes, recovery from complications, and chemotherapy tolerance in an increasingly older and medically complex GO population. Measuring frailty may represent a comprehensive tool for risk prediction. Our study will 1) help fill the current knowledge gaps; 2) translate into changes in clinical practice, locally, in Canada and potentially worldwide; and 3) inform future studies on shared decision-making and interventions.

Figures

Figure 1: Study Flow Diagram

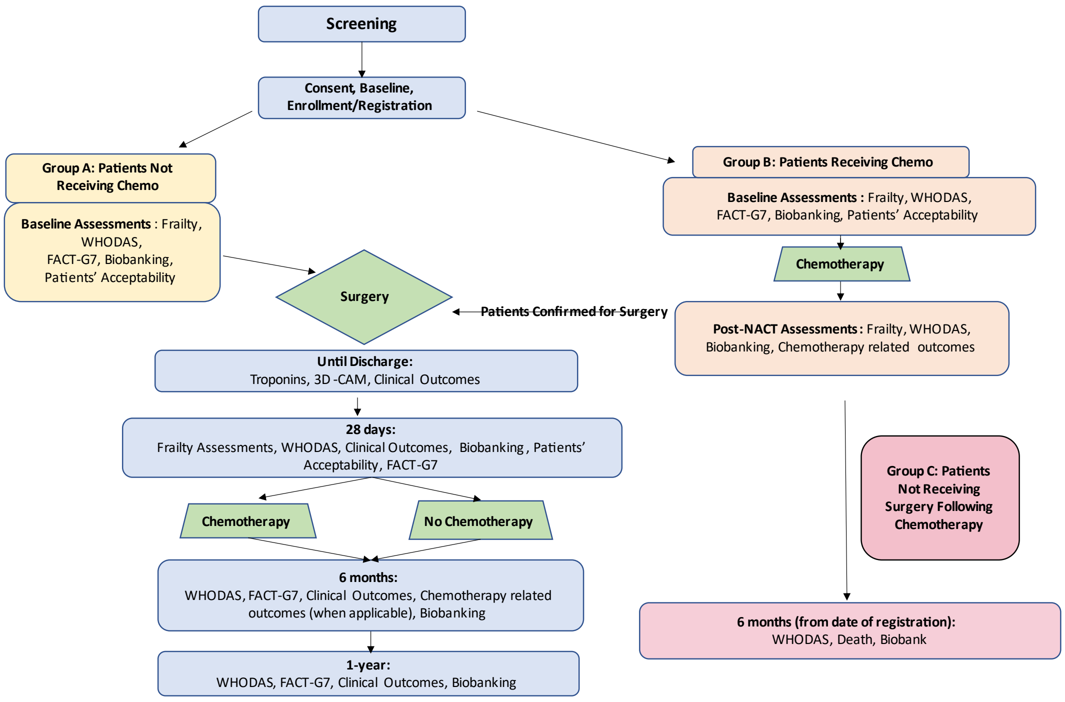

Figure 2 - Study timeline, measurements and data collection

	Before surgery			After surgery			
	Patients Not Receiving Chemotherapy	Patients Receiving Chemotherapy		All Patients			
	Pre-operative Assessment[1]	Before chemotherapy[2,3]	After chemotherapy[2,4]	Day 0 to +3 (+14 days)	28-Day Visit[5]	6-Month Visit[5]	1-Year Visit[5]
**Patient Consent**	X	X					
**Baseline Sociodemographic and Medical History**	X[5]	X[5]					
**Frailty Phenotype**	X	X	X		X		
**Clinical Frailty Scale**	X	X	X		X		
**Troponin Measurement**				X			
**Disability Assessment (WHODAS 2.0)**	X[5]	X[5]	X[5]		X	X	X
**Delirium Assessment (3D-CAM)**				X[6]			
**Health-Related Quality of Life Questionnaire (FACT-G7)**	X	X			X	X	X
**Clinical Outcomes**				X	X	X	X
**Chemotherapy-related outcomes**			X			X	
**Blood Drawing for Biobanking**	X	X	X		X	X	X

[1] Should be done within 45 days prior to surgery
[2] Refers to the round of chemotherapy closest to the surgery date
[3] Should be done within 45 days prior to chemotherapy
[4] Should be done at least 18 days from the last infusion prior to surgery and within 45 days prior to surgery
[5] Can be done in-person or by telephone/videoconference
[6] Should be done twice a day

Appendices

Appendix I. Sample size calculations

The sample size calculation is based on the comparison of two receiver operator characteristic curves (ROC), 2-sided test, with alpha=0.05. The assumed AUC for the RCRI, age, and MINS model is 0.55 (AUC1). The assumed AUC of the frailty assessment model is between 0.65-0.75 (AUC2). The assumed AUC difference is approximately 0.13. We expect an incidence of the primary outcome (all-cause death or disability at 6 months after surgery) to be 20%.

A sample size of 250 patients will give us at least 80% of power to detect a difference of at least 0.130 in the AUC for a model including only the FP-frailty assessment, compared with the AUC for the model including preoperative RCRI and age combined with MINS.

The assumed correlation between the two predictive models is 0.50 for the positive group (those experiencing the outcome) and 0.50 for the negative group (those not experiencing the outcome)

We will include 280 patients to conservatively account for ≤10% who will be lost before or during surgery, or in whom biomarker measurement will not be possible.

Software: PASS 13 (NCSS, LLC. Kaysville, Utah, USA. www.ncss.com)

Sample size calculation

AUC1	AUC difference	AUC2	Target power	Sample size
0.55	0.10	0.65	80%	405
0.55	0.10	0.65	85%	465
0.55	0.10	0.65	90%	545
0.55	0.13	0.68	80%	240
0.55	0.13	0.68	85%	280
0.55	0.13	0.68	90%	325
0.55	0.15	0.70	80%	185
0.55	0.15	0.70	85%	210
0.55	0.15	0.70	90%	245
0.60	0.10	0.70	80%	425
0.60	0.10	0.70	85%	485
0.60	0.10	0.70	90%	570
0.60	0.13	0.73	80%	250
0.60	0.13	0.73	85%	290
0.60	0.13	0.73	90%	340
0.60	0.15	0.75	80%	190
0.60	0.15	0.75	85%	220
0.60	0.15	0.75	90%	255

Appendix II. Clinical outcomes definitions

The following list provides the definitions for the clinical outcomes for which the predictive ability of perioperative frailty and cardiovascular risk assessments will be assessed in the study.

New disability (at 28 days, 6 months, or 1 year after surgery)
Disability status is determined using the 12-item World Health Organization Disability Assessment Schedule (WHODAS 2.0). New disability at any time point is based according to the following criteria that account for baseline disability scores:

1. For individuals with a disability score of <25% at baseline, new disability is defined as a disability score ≥25% at follow-up;
2. For individuals with a disability score ≥25%, new disability is defined an increase in disability score of ≥8%.

Definition of new disability at 28 days, 6 months, and 1 year after surgery will use as baseline disability score the WHODAS 2.0 score measured before surgery.

Sub-classification of death
Vascular death is defined as any death with a vascular cause and includes those deaths following a myocardial infarction, cardiac arrest, stroke, cardiac revascularization procedure (i.e., percutaneous coronary intervention [PCI] or coronary artery bypass graft [CABG] surgery), pulmonary embolus, hemorrhage, or deaths due to an unknown cause. Non-vascular death is defined as any death due to a clearly documented non-vascular cause (e.g., trauma, infection, malignancy).

Oncologic outcomes (at 6 and 12 months)
They will include:
1. PFS, defined as the time from treatment initiation to tumor progression or recurrence or death from any cause, or to the date of censoring at the last time the subject was known to be alive.
2. Cancer-specific death is death directly attributable to the primary gynecological cancer or directly related to its treatment, in the absence of other causes of death.

For the purpose of this study and the evaluation of frailty predictive performance, treatment initiation will be first defined as the time of surgery, and the evaluated frailty assessment will be the one done before surgery (even in patients who had NACT). Secondarily, treatment initiation will be defined as initiation of any cancer-specific treatment, whether NACT or surgery, and the evaluated frailty assessment will be the one done before the first cancer-specific treatment, whether NACT or surgery. Cancer progression/recurrence will be defined as a measurable progression/recurrence documented on imaging.

Chemotherapy-related outcomes
They will include the following, measured at the end of any chemotherapy treatment (whether neo-adjuvant or adjuvant) when considered terminated:
1. Total dose received, defined as Relative Dose Intensity (RDI), as calculated as the percentage of the standard dose that was administered, using the formula below:
   Carboplatin RDI (%) = total dose administered / total standard dose *100
   Paclitaxel RDI (%) = total dose administered / total standard dose *100
2. Time to completion of all chemotherapy cycles, expressed as number of days.
3. Patient's decisional regret, defined as "distress or remorse after a (health care) decision," assessed using the Decisional Regret scale, a validated a 5-item scale (Med Decis Making 2003; 23:281–292)
4. Change in health-related function or well-being, defined as the difference in WHODAS 2.0 score after chemotherapy compared to before chemotherapy.
5. Change in health-related quality of life, defined as the difference in FACT-G7 score after chemotherapy compared to before chemotherapy.

Major vascular complications (at 28 days, 6 months, and 1 year after surgery)
It is defined as a composite of vascular death, and non-fatal myocardial infarction (or myocardial injury for the 28-day time point), stroke, symptomatic proximal venous thromboembolism, and cardiac arrest. The definitions for the components of the composite are the following:

Myocardial Injury after Noncardiac Surgery (MINS)
MINS is defined as any myocardial infarction (as defined below), and any elevated troponin (higher than the local lab threshold) judged to be due to myocardial ischemia (i.e., without evidence of a non-ischemic etiology [e.g. chronic elevation, PE, sepsis, cardioversion]) that occurred within the first 28 days after the initiation of surgery. The only exceptions to the definition of an elevated troponin will be to use a higher threshold for TnT of ≥30 ng/L, and for hsTnT of 20 to <65 ng/L with an absolute change of at least 5 ng/L or an hsTnT level ≥65 ng/L. These thresholds for TnT and hsTnT are based upon data from a large international prospective perioperative cohort study that established troponin thresholds that were independently associated with 30-day mortality after noncardiac surgery.

Myocardial Infarction

If the diagnostic criteria for myocardial infarction includes an elevated troponin, then the definition of MINS must be met to fulfill the diagnostic criteria for myocardial infarction (4th universal definition). The diagnosis of myocardial infarction requires any one of the following criteria.

1. Detection of a rise or fall of a cardiac biomarker (preferably troponin) with at least one value above the 99th percentile of the upper reference limit (URL) together with evidence of myocardial ischemia with at least one of the following:

A. ischemic signs or symptoms (i.e., chest, arm, neck, or jaw discomfort; shortness of breath, pulmonary edema);
B. development of pathologic Q waves present in any two contiguous leads that are ≥ 30 milliseconds;
C. new or presumed ECG changes indicative of ischemia (i.e., ST segment elevation [≥ 2 mm in leads V_1, V_2, or V_3 OR ≥ 1 mm in the other leads], ST segment depression [≥ 1 mm], or symmetric inversion of T waves ≥ 1 mm) in at least two contiguous leads;
D. new left bundle branch block (LBBB); or
E. new cardiac wall motion abnormality on echocardiography or new fixed defect on radionuclide imaging
F. identification of intracoronary thrombus on angiography or autopsy

2. Cardiac death, with symptoms suggestive of myocardial ischemia and presumed new ischemic ECG changes or new LBBB, but death occurred before cardiac biomarkers were obtained, or before cardiac biomarker values would be increased.

3. PCI related myocardial infarction is defined by elevation of a troponin value (>5 x 99th percentile URL) in patients with a normal baseline troponin value (≤99th percentile URL) or a rise of a troponin measurement >20% if the baseline values are elevated and are stable or falling. In addition, either (i) symptoms suggestive of myocardial ischemia or (ii) new ischemic ECG changes or (iii) angiographic findings consistent with a procedural complication or (iv) imaging demonstration of new loss of viable myocardium or new regional wall motion abnormality are required.

4. Stent thrombosis associated with myocardial infarction when detected by coronary angiography or autopsy in the setting of myocardial ischemia and with a rise and/or fall of cardiac biomarker values with at least one of value above the 99th percentile URL.

5. CABG related myocardial infarction is defined by elevation of cardiac biomarker values (>10 x 99th percentile URL) in patients with a normal baseline troponin value (≤99th percentile URL). In addition, either (i) new pathological Q waves or new LBBB, or (ii) angiographic documented new graft or new native coronary artery occlusion, or (iii) imaging evidence of new loss of viable myocardium or new regional wall motion abnormality.

6. For patients who are believed to have suffered a myocardial infarction within 28 days of a MINS event or within 28 days of a prior myocardial infarction, the following criterion for myocardial infarction is required:
Detection of a rise or fall of a cardiac biomarker (preferably troponin) with at least one value above the 99th percentile of the URL and 20% higher than the last troponin measurement related to the preceding event together with evidence of myocardial ischemia with at least one of the following:

A. ischemic signs or symptoms (i.e., chest, arm, neck, or jaw discomfort; shortness of breath, pulmonary edema);
B. development of pathologic Q waves present in any two contiguous leads that are ≥ 30 milliseconds;
C. new or presumed new ECG changes indicative of ischemia (i.e., ST segment elevation [≥ 2 mm in leads V_1, V_2, or V_3 OR ≥ 1 mm in the other leads], ST segment depression [≥ 1 mm], or symmetric inversion of T waves ≥ 1 mm) in at least two contiguous leads;
D. new LBBB; or
E. new cardiac wall motion abnormality on echocardiography or new fixed defect on radionuclide imaging
F. identification of intracoronary thrombus on angiography or autopsy

Stroke
Stroke is defined as a new focal neurological deficit thought to be vascular in origin with signs or symptoms lasting more than 24 hours or leading to death.

Symptomatic Proximal Venous Thromboembolism
Venous thromboembolism that includes symptomatic PE or symptomatic proximal deep vein thrombosis

Symptomatic Pulmonary Embolism (PE)
The diagnosis of symptomatic PE requires symptoms (e.g., dyspnea, pleuritic chest pain) or signs (e.g., hypoxia, increased work of breathing) and any one of the following:

1. A high probability ventilation/perfusion lung scan,
2. An intraluminal filling defect of segmental or larger artery on a helical CT scan,
3. An intraluminal filling defect on pulmonary angiography, or
4. A positive diagnostic test for DVT (e.g., positive compression ultrasound) and one of the following:
   A. non-diagnostic (i.e., low or intermediate probability) ventilation/perfusion lung scan, or
   B. non-diagnostic (i.e., subsegmental defects or technically inadequate study) helical CT scan

Symptomatic Proximal Deep Venous Thrombosis (DVT)
The diagnosis of symptomatic proximal DVT requires:

1. symptoms or signs that suggest DVT (e.g., leg pain or swelling),
2. thrombosis involving the popliteal vein or more proximal veins for leg DVT OR axillary or more proximal veins for arm DVTs

Any of the following defines evidence of vein thrombosis:

A. a persistent intraluminal filling defect on contrast venography (including on computed tomography),
B. noncompressibility of one or more venous segments on B mode compression ultrasonography, or
C. A clearly defined intraluminal filling defect on doppler imaging in a vein that cannot have compressibility assessed (e.g., iliac, inferior vena cava, subclavian).

Nonfatal cardiac arrest
Nonfatal cardiac arrest is defined as successful resuscitation from either documented or presumed ventricular fibrillation, sustained ventricular tachycardia, asystole, or pulseless electrical activity requiring cardiopulmonary resuscitation, pharmacological therapy, or cardiac defibrillation.

Infection, and infection with sepsis (28 days, 6 months, and 1 year)
Infection is defined as a pathologic process caused by the invasion of normally sterile tissue or fluid or body cavity by pathogenic or potentially pathogenic organisms.

The Third International Consensus Definitions Task Force defines sepsis as a "life-threatening organ dysfunction due to a dysregulated host response to infection." Based on the Third International Consensus Definitions for Sepsis and Septic Shock (Sepsis-3) criteria, sepsis will require a quick Sequential Organ Failure Assessment (qSOFA) Score ≥2 points due to infection. The qSOFA includes the following items and scoring system:
1. Altered mental status (1 point)
2. systolic blood pressure of 100 mm Hg or less (1 point), and
3. respiratory rate of 22 breaths/min or more (1 point).

In-hospital delirium
Delirium during the first 3 days after surgery or before discharge from the hospital, based on Confusion Assessment Method (CAM). According to CAM, patients are diagnosed with delirium if they meet the first 2 criteria (acute onset with fluctuating course, AND attention deficit), and at least one of the second 2 criteria (disorganized thinking OR altered level of consciousness). Participants will be screened for postoperative delirium while in hospital, twice daily, during the first 3 days after surgery or until discharge (if before 3 days), by research personnel, using the 3D-CAM, or the CAM-ICU any time the participants are in the PACU or in ICU.

Bleeding Independently Associated with Mortality after noncardiac Surgery (BIMS)

BIMS is a bleeding meeting any of the following 3 criteria:
1. Leading to a postoperative hemoglobin <70 g/L
2. Requiring transfusion of one or more units of red blood cells
3. Judged to be the immediate cause of death

New clinically important atrial fibrillation

The definition of new clinically important atrial fibrillation requires the documentation of atrial fibrillation or atrial flutter of any duration on an ECG or rhythm strip, which results in angina congestive heart failure, symptomatic hypotension, or requires treatment with a rate controlling drug, antiarrhythmic drug, or electrical cardioversion.

Acute congestive heart failure

The definition of congestive heart failure requires at least one of the following clinical signs (i.e., any of the following signs: elevated jugular venous pressure, respiratory rales/crackles, crepitations, or presence of S3) and at least one of the following:

1. Radiographic findings (i.e., vascular redistribution, interstitial pulmonary edema, or frank alveolar pulmonary edema) OR

2. Heart failure treatment implemented with diuretics with documented clinical improvement.

Appendix III – Measuring acceptability of frailty assessment

Study personnel will invite a convenience sample of consenting physicians involved in the perioperative clinical assessment of the study population (e.g., the Gynecologic Oncology group, the Perioperative Medicine Service, etc.), with no experience with frailty tools, to participate in the administration of the FP and CSF; then the study personnel will administer the acceptability questionnaire to the consenting physicians. Patient's acceptability of frailty assessments will be evaluated administering a questionnaire in a purposeful sample of the main study participants.

Physicians' acceptability

A convenience sample of consenting physicians involved in the perioperative clinical assessment of the study population (e.g., the Gynecologic Oncology group, the Perioperative Medicine Service, etc.), with no experience with frailty tools, will be invited to participate in the administration of each frailty instrument (FP and CFS). They will be then administered the following questionnaire by research personnel. The questionnaire is a modified version of the OADRI where only items deemed relevant are retained:

The tool is easy to use
The tool is useful in my practice.
The wording of the tool is clear and unambiguous.
My colleagues support use of the tool.
Patients benefit from use of the tool.
Using the tool results in improved use of resources.
The evidence supporting the tool is flawed.
I'm already using another tool or similar strategy.
The tool does not account for an important clinical cue.
The environment I work in makes it difficult to use the tool.

Physicians are asked to rate each item, for each tool, along a 7-point Likert scale including the following options: strongly disagree, moderately disagree, slightly disagree, no opinion/don't know, slightly agree, moderately agree, strongly agree.

Patients' acceptability

The first 10 patients at each site will be asked about the acceptability of the Frailty Phenotype Assessment and WHODAS questionnaire shortly following their completion at the baseline study visit. The next 10 patients (different from those asked at baseline) will be asked about the acceptability of the Frailty Phenotype and WHODAS questionnaire shortly following their completion at the 28-day follow-up visit. In total, 20 patients at each site will be asked about the acceptability of the FP and WHODAS, 10 at each indicated visit. If additional patients are required to be asked the acceptability questionnaire, the PO office will contact the site to indicate how many additional patients should be asked.

1. Was the patient asked about the acceptability of the Frailty Phenotype Assessment?	No<br>Yes
2. On a scale of 0 to 5, with 0 being not easy at all, and 5 being the easiest, how easy was it to complete the Frailty Phenotype assessment?	0<br>1<br>2<br>3<br>4<br>5<br>No opinion<br>Don't know
3. The Frailty Phenotype Assessment took a long time to complete	Strongly disagree<br>Disagree<br>Neither Disagree or Agree<br>Agree<br>Strongly Agree
4. The language used in the Frailty Phenotype Assessment was easy to understand	Strongly disagree<br>Disagree<br>Neither Disagree or Agree<br>Agree<br>Strongly Agree

5. One or more tasks of the Frailty Phenotype Assessment was/were physically or psychologically/emotionally burdensome to complete	Strongly disagree<br>Disagree<br>Neither Disagree or Agree<br>Agree<br>Strongly Agree
6. On a scale of 0 to 5, with 0 being not helpful at all, and 5 being the most helpful, how helpful for understanding your health was it to complete the Frailty Phenotype assessment?	0<br>1<br>2<br>3<br>4<br>5<br>No Opinion<br>Don't Know
7. Would you complete the Frailty Phenotype assessment again?	Yes<br>No

1. Was the patient asked about the acceptability of WHODAS?	No<br><br>Yes
2. On a scale of 0 to 5, with 0 being not easy at all, and 5 being the easiest, how easy was it to complete the WHODAS questionnaire?	0<br>1<br>2<br>3<br>4<br>5<br>No opinion<br>Don't know
3. The WHODAS Assessment took a long time to complete	Strongly disagree<br>Disagree<br>Neither Disagree or Agree<br>Agree<br>Strongly Agree
4. The language used in the WHODAS assessment was easy to understand	Strongly disagree<br>Disagree<br>Neither Disagree or Agree<br>Agree<br>Strongly Agree
5. One or more items of the WHODAS questionnaires was/were psychologically/emotionally burdensome to complete	Strongly disagree<br>Disagree<br>Neither Disagree or Agree<br>Agree<br>Strongly Agree
6. On a scale of 0 to 5, with 0 being not helpful at all, and 5 being the most helpful, how helpful for understanding your health was it to complete the WHODAS questionnaire?	0<br>1<br>2<br>3<br>4<br>5<br>No Opinion<br>Don't Know
7. Would you complete the WHODAS questionnaire again?	Yes<br>No

References

1. Kadambi S, Loh KP, Dunne R, et al. Older adults with cancer and their caregivers - current landscape and future directions for clinical care. Nat Rev Clin Oncol 2020;17(12):742-755. DOI: 10.1038/s41571-020-0421-z.
2. Dumas L, Ring A, Butler J, Kalsi T, Harari D, Banerjee S. Improving outcomes for older women with gynaecological malignancies. Cancer treatment reviews 2016;50:99-108. (In eng). DOI: 10.1016/j.ctrv.2016.08.007.
3. Narasimhulu DM, McGree ME, Weaver AL, et al. Frailty is a determinant of suboptimal chemotherapy in women with advanced ovarian cancer. Gynecol Oncol 2020;158(3):646-652. DOI: 10.1016/j.ygyno.2020.05.046.
4. Moore KN, Reid MS, Fong DN, et al. Ovarian cancer in the octogenarian: does the paradigm of aggressive cytoreductive surgery and chemotherapy still apply? Gynecol Oncol 2008;110(2):133-9. DOI: 10.1016/j.ygyno.2008.03.008.
5. Lee YY, Lee JW, Lu L, et al. Impact of interval from primary cytoreductive surgery to initiation of adjuvant chemotherapy in advanced epithelial ovarian cancer. Int J Gynaecol Obstet 2018;143(3):325-332. DOI: 10.1002/ijgo.12653.
6. Armstrong DK, Bundy B, Wenzel L, et al; Gynecologic Oncology Group. Intraperitoneal cisplatin and paclitaxel in ovarian cancer. N Engl J Med. 2006;354:34-43.
7. Falandry C, Rousseau F, Mouret-Reynier MA, et al. Efficacy and Safety of First-line Single-Agent Carboplatin vs Carboplatin Plus Paclitaxel for Vulnerable Older Adult Women With Ovarian Cancer: A GINECO/GCIG Randomized Clinical Trial. JAMA Oncol 2021;7(6):853-861. DOI: 10.1001/jamaoncol.2021.0696.
8. May T, Altman A, McGee J, et al. Examining Survival Outcomes of 852 Women With Advanced Ovarian Cancer: A Multi-institutional Cohort Study. Int J Gynecol Cancer 2018;28(5):925-931. DOI: 10.1097/IGC.0000000000001244.
9. Langstraat C, Cliby WA. Considerations in the surgical management of ovarian cancer in the elderly. Curr Treat Options Oncol 2013;14(1):12-21. DOI: 10.1007/s11864-012-0216-2.
10. Lee TH, Marcantonio ER, Mangione CM, et al. Derivation and prospective validation of a simple index for prediction of cardiac risk of major noncardiac surgery. Circulation 1999;100(10):1043-9. DOI: 10.1161/01.cir.100.10.1043.
11. Ford MK, Beattie WS, Wijeysundera DN. Systematic review: prediction of perioperative cardiac complications and mortality by the revised cardiac risk index. Ann Intern Med 2010;152(1):26-35. DOI: 10.7326/0003-4819-152-1-201001050-00007.
12. Duceppe E, Parlow J, MacDonald P, et al. Canadian Cardiovascular Society Guidelines on Perioperative Cardiac Risk Assessment and Management for Patients Who Undergo Noncardiac Surgery. Can J Cardiol 2017;33(1):17-32. DOI: 10.1016/j.cjca.2016.09.008.
13. Writing Committee for the VISION Study Investigators. Association of postoperative high-sensitivity troponin levels with myocardial injury and 30-day mortality among patients undergoing noncardiac surgery. JAMA 2017;317(16):1642-1651. DOI: 10.1001/jama.2017.4360.
14. Vascular Events In Noncardiac Surgery Patients Cohort Evaluation Study I, Devereaux PJ, Chan MT, et al. Association between postoperative troponin levels and 30-day mortality among patients undergoing noncardiac surgery. JAMA 2012;307(21):2295-304. DOI: 10.1001/jama.2012.5502.
15. Vascular Events in Noncardiac Surgery Patients Cohort Evaluation (VISION) Study Investigators. Association between complications and death within 30 days after noncardiac surgery. CMAJ 2019;191(30):E830-E837. DOI: 10.1503/cmaj.190221.
16. Smilowitz NR, Redel-Traub G, Hausvater A, et al. Myocardial Injury After Noncardiac Surgery: A Systematic Review and Meta-Analysis. Cardiol Rev 2019;27(6):267-273. DOI: 10.1097/CRD.0000000000000254.
17. Fried LP, Ferrucci L, Darer J, Williamson JD, Anderson G. Untangling the concepts of disability, frailty, and comorbidity: implications for improved targeting and care. J Gerontol A Biol Sci Med Sci 2004;59(3):255-63. DOI: 10.1093/gerona/59.3.m255.
18. Song X, Mitnitski A, Rockwood K. Prevalence and 10-year outcomes of frailty in older adults in relation to deficit accumulation. J Am Geriatr Soc 2010;58(4):681-7. DOI: 10.1111/j.1532-5415.2010.02764.x.
19. Hanlon P, Nicholl BI, Jani BD, Lee D, McQueenie R, Mair FS. Frailty and pre-frailty in middle-aged and older adults and its association with multimorbidity and mortality: a prospective

analysis of 493 737 UK Biobank participants. Lancet Public Health 2018;3(7):e323-e332. DOI: 10.1016/S2468-2667(18)30091-4.
20. Rockwood K, Song X, Mitnitski A. Changes in relative fitness and frailty across the adult lifespan: evidence from the Canadian National Population Health Survey. CMAJ 2011;183(8):E487-94. DOI: 10.1503/cmaj.101271.
21. McIsaac DI, Taljaard M, Bryson GL, et al. Frailty as a Predictor of Death or New Disability After Surgery: A Prospective Cohort Study. Ann Surg 2020;271(2):283-289. DOI: 10.1097/SLA.0000000000002967.
22. Ethun CG, Bilen MA, Jani AB, Maithel SK, Ogan K, Master VA. Frailty and cancer: Implications for oncology surgery, medical oncology, and radiation oncology. CA Cancer J Clin 2017;67(5):362-377. DOI: 10.3322/caac.21406.
23. Chesney TR, Haas B, Coburn N, et al. Association of frailty with long-term homecare utilization in older adults following cancer surgery: Retrospective population-based cohort study. Eur J Surg Oncol 2021;47(4):888-895. DOI: 10.1016/j.ejso.2020.09.009.
24. McIsaac DI, Beaule PE, Bryson GL, Van Walraven C. The impact of frailty on outcomes and healthcare resource usage after total joint arthroplasty: a population-based cohort study. Bone Joint J 2016;98-B(6):799-805. DOI: 10.1302/0301-620X.98B6.37124.
25. McIsaac DI, Bryson GL, van Walraven C. Association of Frailty and 1-Year Postoperative Mortality Following Major Elective Noncardiac Surgery: A Population-Based Cohort Study. JAMA Surg 2016;151(6):538-45. DOI: 10.1001/jamasurg.2015.5085.
26. Ngo-Huang A, Holmes HM, des Bordes JKA, et al. Association between frailty syndrome and survival in patients with pancreatic adenocarcinoma. Cancer Med 2019;8(6):2867-2876. DOI: 10.1002/cam4.2157.
27. Shahrokni A, Tin A, Alexander K, et al. Development and Evaluation of a New Frailty Index for Older Surgical Patients With Cancer. JAMA Netw Open 2019;2(5):e193545. DOI: 10.1001/jamanetworkopen.2019.3545.
28. Dai S, Yang M, Song J, Dai S, Wu J. Impacts of Frailty on Prognosis in Lung Cancer Patients: A Systematic Review and Meta-Analysis. Frontiers in Medicine 2021;8(1174) (Systematic Review) (In English). DOI: 10.3389/fmed.2021.715513.
29. Fried LP, Tangen CM, Walston J, et al. Frailty in older adults: evidence for a phenotype. J Gerontol A Biol Sci Med Sci 2001;56(3):M146-56. DOI: 10.1093/gerona/56.3.m146.
30. Searle SD, Mitnitski A, Gahbauer EA, Gill TM, Rockwood K. A standard procedure for creating a frailty index. BMC Geriatr 2008;8:24. DOI: 10.1186/1471-2318-8-24.
31. Gobbens RJ, van Assen MA, Luijkx KG, Wijnen-Sponselee MT, Schols JM. The Tilburg Frailty Indicator: psychometric properties. J Am Med Dir Assoc 2010;11(5):344-55. DOI: 10.1016/j.jamda.2009.11.003.
32. Rolfson DB, Majumdar SR, Tsuyuki RT, Tahir A, Rockwood K. Validity and reliability of the Edmonton Frail Scale. Age Ageing 2006;35(5):526-9. DOI: 10.1093/ageing/afl041.
33. SHAW R, GWYTHER H, HOLLAND C, et al. Understanding frailty: Meanings and beliefs about screening and prevention across key stakeholder groups in Europe. Ageing and Society 2018;6(38):1223-1252. DOI: 10.1017/S0144686X17000745.
34. Auyeung TW, Lee JS, Kwok T, Woo J. Physical frailty predicts future cognitive decline - a four-year prospective study in 2737 cognitively normal older adults. J Nutr Health Aging 2011;15(8):690-4. DOI: 10.1007/s12603-011-0110-9.
35. Boyle PA, Buchman AS, Wilson RS, Leurgans SE, Bennett DA. Physical frailty is associated with incident mild cognitive impairment in community-based older persons. J Am Geriatr Soc 2010;58(2):248-55. DOI: 10.1111/j.1532-5415.2009.02671.x.
36. Buchman AS, Yu L, Wilson RS, Schneider JA, Bennett DA. Association of brain pathology with the progression of frailty in older adults. Neurology 2013;80(22):2055-61. DOI: 10.1212/WNL.0b013e318294b462.
37. Kelaiditi E, Cesari M, Canevelli M, et al. Cognitive frailty: rational and definition from an (I.A.N.A./I.A.G.G.) international consensus group. J Nutr Health Aging 2013;17(9):726-34. DOI: 10.1007/s12603-013-0367-2.
38. St John PD, Tyas SL, Griffith LE, Menec V. The cumulative effect of frailty and cognition on mortality - results of a prospective cohort study. Int Psychogeriatr 2017;29(4):535-543. DOI: 10.1017/S1041610216002088.
39. Giannini A, Di Donato V, Schiavi MC, May J, Panici PB, Congiu MA. Predictors of postoperative overall and severe complications after surgical treatment for endometrial cancer: The role of the fragility index. Int J Gynaecol Obstet 2020;148(2):174-180. DOI: 10.1002/ijgo.13020.

40. Di Donato V, Di Pinto A, Giannini A, et al. Modified fragility index and surgical complexity score are able to predict postoperative morbidity and mortality after cytoreductive surgery for advanced ovarian cancer. Gynecol Oncol 2021;161(1):4-10. DOI: 10.1016/j.ygyno.2020.08.022.
41. Kumar A, Langstraat CL, DeJong SR, et al. Functional not chronologic age: Frailty index predicts outcomes in advanced ovarian cancer. Gynecol Oncol 2017;147(1):104-109. DOI: 10.1016/j.ygyno.2017.07.126.
42. Courtney-Brooks M, Tellawi AR, Scalici J, et al. Frailty: an outcome predictor for elderly gynecologic oncology patients. Gynecol Oncol 2012;126(1):20-4. DOI: 10.1016/j.ygyno.2012.04.019.
43. de Arruda FN, Oonk MHM, Mourits MJE, de Graeff P, Jalving M, de Bock GH. Determinants of health-related quality of life in elderly ovarian cancer patients: The role of frailty and dependence. Gynecol Oncol 2019;153(3):610-615. DOI: 10.1016/j.ygyno.2019.03.249.
44. Mah SJ, Anpalagan T, Marcucci M, Eiriksson L, Reade CJ, Jimenez W, et al. The five-factor modified frailty index predicts adverse postoperative and chemotherapy outcomes in gynecologic oncology. Gynecol Oncol. 2022;166(1):154-61.
45. Ashar FN, Moes A, Moore AZ, et al. Association of mitochondrial DNA levels with frailty and all-cause mortality. J Mol Med (Berl) 2015;93(2):177-186. DOI: 10.1007/s00109-014-1233-3.
46. Andreux PA, van Diemen MPJ, Heezen MR, et al. Mitochondrial function is impaired in the skeletal muscle of pre-frail elderly. Sci Rep 2018;8(1):8548. DOI: 10.1038/s41598-018-26944-x.
47. Picca A, Calvani R, Cesari M, et al. Biomarkers of Physical Frailty and Sarcopenia: Coming up to the Place? Int J Mol Sci 2020;21(16). DOI: 10.3390/ijms21165635.
48. Picca A, Beli R, Calvani R, et al. Older Adults with Physical Frailty and Sarcopenia Show Increased Levels of Circulating Small Extracellular Vesicles with a Specific Mitochondrial Signature. Cells 2020;9(4). DOI: 10.3390/cells9040973.
49. Lee TH, Marcantonio ER, Mangione CM, et al. Derivation and prospective validation of a simple index for prediction of cardiac risk of major noncardiac surgery. Circulation 1999;100:1043-9.
50. Andrews G, Kemp A, Sunderland M, Von Korff M, Ustun TB. Normative data for the 12 item WHO Disability Assessment Schedule 2.0. PLoS One 2009;4(12):e8343. DOI: 10.1371/journal.pone.0008343.
51. Brehaut JC, Graham ID, Wood TJ, et al. Measuring acceptability of clinical decision rules: validation of the Ottawa acceptability of decision rules instrument (OADRI) in four countries. Med Decis Making. 2010;30(3):398-408. doi: 10.1177/0272989X09344747.
52. DeLong ER, DeLong DM, Clarke-Pearson DL. Comparing the areas under two or more correlated receiver operating characteristic curves: a nonparametric approach. Biometrics 1988;44(3):837-45. (https://www.ncbi.nlm.nih.gov/pubmed/3203132).
53. Alba AC, Agoritsas T, Walsh M, et al. Discrimination and Calibration of Clinical Prediction Models: Users' Guides to the Medical Literature. JAMA 2017;318(14):1377-1384. DOI: 10.1001/jama.2017.12126.
